# Comparative performances of prognostic indexes for breast cancer patients presenting with brain metastases

**DOI:** 10.1186/1471-2407-13-70

**Published:** 2013-02-08

**Authors:** Antoine-Laurent Braccini, David Azria, Simon Thezenas, Gilles Romieu, Jean-Marc Ferrero, William Jacot

**Affiliations:** 1 Department of Radiation Oncology, Val d’Aurelle Cancer Institute, 208 rue des apothicaires, Montpellier 34298, France; 2 Department of Biostatistics, Val d’Aurelle Cancer Institute, 208 rue des apothicaires, 34298, Montpellier, France; 3 Department of Medical Oncology, Val d’Aurelle Cancer Institute, 208 rue des apothicaires, 34298, Montpellier, France; 4 Department of Medical Oncology, Antoine Lacassagne Cancer Institute, 33, avenue de Valombrose, 06189, Nice Cedex 02, France

**Keywords:** Breast cancer, Brain metastases, Prognostic indexes, Biological subtype

## Abstract

**Background:**

Several prognostic indexes (PI) have been developed in the brain metastases (BM) setting to help physicians tailor treatment options and stratify patients enrolled in clinical studies. The aim of our study was to compare the clinical relevance of the major PI for breast cancer BM.

**Methods:**

Clinical and biological data of 250 breast cancer patients diagnosed with BM at two institutions between 1995 and 2010 were retrospectively reviewed. The prognostic value and accuracy of recursive partitioning analysis (RPA), graded prognostic assessment (GPA), basic score for BM (BS-BM), breast RPA, breast GPA, Le Scodan’s Score and a clinico-biological score developed in a phase I study (P1PS) were assessed using Cox regression models. PI comparison was performed using Harrell’s concordance index.

**Results:**

After a median follow-up of 4.5 years, median overall survival (OS) from BM diagnosis was 8.9 months (CI 95%, 6.9–10.3 months). All PI were significantly associated with OS. Harrell’s concordance indexes C favored BS-BM and RPA. In multivariate analysis, the RPA, Le Scodan’s score and GPA were found to be the best independent predictors of OS. In multivariate analysis restricted to the 159 patients with known LDH and proteinemia, RPA 2 and 3, Le Scodan’s Score 3 and P1PS 2/3 were associated with worse survival. RPA was the most accurate score to identify patients with long (superior to 12 months) and short (inferior to 3 months) life expectancy.

**Conclusions:**

RPA seems to be the most useful score and performs better than new PI for breast cancer BM.

## Background

The Recursive Partitioning Analysis RPA [1] was the first prognostic score developed in the brain metastases (BM) setting. This classification was created in 1997 by the Radiation Therapy Oncology Group after analysis of the relative contributions of pretreatment variables to survival of patients with BM. Since this date, several scores and prognostic indexes (PI), such as the Graded Prognosis Assessment (GPA) [2], the Basic Score for BM (BS-BM) [3], the Phase 1 Prognostic Score (P1PS) [4], the Rotterdam score [5], the Score Index for Radiosurgery (SIR) [6] and the Rades’s score [7] have been developed both to help physicians tailor treatment options depending on patient prognosis, and to stratify patients enrolled in clinical studies. However, it has been demonstrated that the prognostic value of these scoring systems differs according to the primary tumor site [8], which raises the question of the usefulness of a breast-specific score.

Breast cancer is the second cause of BM, after lung cancer. Breast cancer is a heterogeneous disease with metastatic pattern and survival varying with the expression of biological markers such as the hormonal receptor (HR) status and human epidermal growth factor receptor-2 (HER2) overexpression. While the incidence of BM from breast cancer has increased over the past decade, especially for the subgroup of HER2-overexpressing tumors, several studies have shown that biological subtypes influence survival, even after BM diagnosis. In a series of 223 breast cancer patients irradiated for BM, Dawood *et al.* showed that HER2 positive status was an independent favorable prognostic factor [9]. On the contrary, the triple negative population seems to be associated with worse prognosis [10,11]. These results have prompted the development of specific prognostic scores for BM from breast cancer taking into account either tumor phenotypic characteristics [12,13] or not [14]. Given the number of scoring systems that have been devised for clinical use, the aim of our study was to compare the clinical relevance of the major existing prognostic scores in a cohort of breast cancer patients with BM and known HER2 and HR status.

## Methods

### Study population

Medical records of breast cancer patients with BM were retrospectively extracted from the databases of two French cancer centers. Patients were accrued over a 15-year period, between 1995 and 2010. Inclusion criteria were as follows: histologically proven breast carcinoma, intradural BM detected by contrast-enhanced cerebral computed tomography or magnetic resonance imaging, and known HR and HER2 status. The tumor was considered HR positive when more than 10% of cells were labeled in immunohistochemistry (IHC) or when the concentrations of estrogen and progesterone receptors were above 10 ng/ml and 50 ng/ml using the radioligand binding method, respectively. The tumor was considered HER2 positive if the primary tumor was scored 3+ by IHC or if the HER2 gene was amplified by fluorescence in situ hybridization (FISH). If the tumor was scored 2+ by IHC, it was re-analyzed using FISH. Patients with history of other primitive carcinoma or leptomeningeal carcinomatosis were excluded. In addition, an additional brain MRI was performed to all patient presenting with 1 to 3 BM at baseline CT-scan. Clinical data and, when available, biological parameters were extracted in order to score patients using the RPA [1], the GPA [2], the BS-BM [3], the P1PS [4], the Breast-GPA [12], the Breast-RPA [14] and Le Scodan’s score [13], whose constituting parameters are detailed in Table 1. Ethical approval, as well as permission to create, complete and access the comprehensive database used in this study, was provided by the local research ethics committee of the Val d’Aurelle Cancer Institute. Due to the retrospective, non interventional nature of this study, no consent was requested by the local research ethics committee.

**Table 1:** Prognostic indexes parameters

**A: Clinical parameters used for 5 prognostic indexes (RPA, GPA, BS-BM, Breast RPA, and Breast GPA).**					
**RPA**					
Class 1	Age <65 y, KPS ≥ 70, controlled primary tumor, no extracranial metastases				
Class 2	All patients not in Class I or III				
Class 3	KPS < 70				
**GPA**					
			0	0,5	1
Age			> 60	50-59	<50
KPS			<70	70-80	90-100
Number of BM			> 3	2-3	1
Extracranial metastases			Yes	-	No
**BS-BM**					
				0	1
KPS				50-70	80-100
Control of primary tumor				No	Yes
Extracranial metastases				Yes	No
**Breast RPA**					
Class 1	1–2 brain metastases and extracranial disease absent or controlled and KPS 100				
Class 2	All patients not in Class I or III				
Class 3	Multiple brain metastases and KPS ≤ 60				
**Breast GPA**					
	0	0,5	1	1.5	2
Age	≥ 60	<60			
KPS	≤ 50	60	70-80	90-100	
Genetic subtype	Basal		Luminal A	HER2	Luminal B
**B: Clinico-biological parameters used for the P1PS and Le Scodan’s prognostic indexes.**					
**P1PS**					
		0		1	
Sites of metastases		0-2		>2	
Serum LDH		<ULN		>ULN	
Albumin, g/L		≥35		<35	
**Le Scodan Score**					
Class I	HER2+ tumors treated with trastuzumab				
Class II	All patients not in Class I or III				
Class III	Tumors not treated with trastuzumab and: lymphopenia at BM diagnosis or KPS < 70 and ≥ 50 years old at BM diagnosis or KPS ≥ 70 and triple negative tumors				

*RPA*, Recursive Partitioning Analysis, *GPA*, Graded Partitioning Analysis, *BS-BM*, Basic Score for Brain Metastases, *BM*, Brain Metastases, *P1PS*, phase 1 prognostic score, *KPS*, Karnofsky Performance Status, *BM*, Brain Metastases, *LDH*, Lactate Dehydrogenase, *ULN*, Upper Limit of Normal.

### Statistical analyses

Categorical variables were reported by means of contingency tables. For continuous variables, median and range values were computed. To investigate the association between study features, univariate statistical analyses were performed using Pearson’s Chi-2 test or Fisher’s exact test if applicable for categorical variables. The Kruskal-Wallis test or Student *T* test were used for continuous variables. Overall survival (OS) time was measured from the date of BM diagnosis to the date of death from any cause. Patients alive without event were censored at the closing date of the study analysis (August 1st, 2011). OS rates and median values were estimated according to the Kaplan-Meier method [15], and presented with their 95% confidence intervals (95% CIs). The median length of follow-up was estimated using a reverse Kaplan-Meier method and presented with 95% CIs.

Pair wise comparisons of subgroups were performed for each score. Survival curves were drawn and the log-rank test was performed to assess differences between groups. Harrell’s concordance Index (C index) was used to assess the discriminating ability of the different PIs [16]. To investigate prognostics factors, multivariate analyses were carried out using the Cox’s proportional hazards regression model with a stepwise selection procedure [17,18]. Hazard ratios (HR) with 95% CIs are presented to display risk reductions. All p values reported are two-sided, and the significance level was set at 5% (p < 0.05). Statistical analysis was performed using the STATA 11 software (Stata Corporation, College Station, TX).

## Results

### Patient characteristics

There were a total of two hundred and fifty patients included in this analysis. Patient characteristics are detailed in Table 2. At the time of BM diagnosis, the median age was 55 years (range 25–85), and 74% of patients had good performance status (80–100). The brain was the first metastatic site in about one third of patients (34%), and the only site of metastatic disease in 12% of patients. Of the 250 patients, 44% had a primary tumor that over-expressed HER2, while 26% were diagnosed with a triple negative breast cancer (negative HR and HER2 status). A total of 47 patients (18.8%) underwent targeted local treatment, namely stereotactic radiotherapy or surgery. Whole brain radiation therapy (WBRT), used as primary treatment but also as adjuvant treatment after localized treatment, was given to 217 patients (86.8%). Fifteen patients received best supportive care only. After a median follow-up of 4.5 years, the median OS (MOS) was 8.9 months (95% CI, 6.9-10.3 months). The six-month, one-year and two-year overall survival rates were 61% (95% CI, 54-67%), 40% (95% CI, 34-46%) and 22% (95% CI, 17-27%), respectively.

**Table 2:** Study population

**Patient characteristics**	**Number of patients**	**%**
Age at breast cancer diagnosis (years)		
Median, range	50 (23–82)	
Age at BM diagnosis (years)		
Median, range	55 (25–85)	
Time between initial diagnosis and BM diagnosis (months)		
Median, range	39.4 (0–319.2)	
Hormone receptor status		
Positive	119	47.6
Negative	131	52.4
HER-2 status		
Positive	109	43.6
Negative	141	56.4
Karnofsky Performance status		
100	66	26.4
80–90	119	47.6
60–70	28	11.2
40–50	32	12.8
10-30	5	2
Extra-cerebral metastases at BM diagnosis		
Yes	219	87.6
No	31	12.4
Systemic treatment after BM diagnosis		
Chemotherapy		
Yes	177	70.8
No	73	29.2
Anti-HER2 treatment for HER2+ patients		
Yes	20	18.3
No	89	81.7

*BM*, Brain Metastases, *KPS*, Karnofsky Performance Status.

### Prognostic indexes analysis

**Figure 1:**
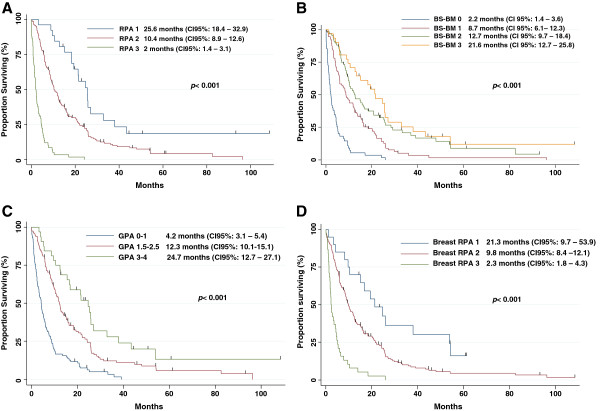
Overall survival according to (A) the RPA, (B) the BS-BM, (C) the GPA, (D) the Breast RPA, (E) the Breast GPA, (F) Le Scodan’s score, (G) the P1PS score.

Table 3 lists the study population distribution as well as the MOS for each PI. Survival curves are depicted in Figure 1. The results showed that all scores were able to discriminate with statistical significance (p < 0.001) patients for OS according to the prognostic category. MOS times for the RPA classes I, II and III were 25.6 months (95% CI 18.4-32.9), 10.4 months (95% CI 8.9-12.6), and 2 months (95% CI 1.4-3.1), respectively. For the GPA classes I, II and III, the MOS were 25.6 months (95% CI 3.1-5.4), 12.3 months (95% CI 10.1-15.1), and 24.7 months (95% CI 12.7-27.1), respectively. In patients stratified in the classes I, II, III and IV using the BS-BM prognostic scores, the MOS were 2.2 months (95% CI 1.4-3.6), 8.7 months (95% CI 6.1-12.3), 12.7 months (95% CI 12.7-27.1), and 21.6 months (95% CI 12.7-25.6), respectively. With respect to the P1PS, ninety-one patients could not be classified due to missing biological data. The MOS were 16.4 months (95% CI 11.9-23.3) vs. 5.9 months (95% CI 3.4-8.4) in remaining patients with P1PS scores of 0/1 vs. 2/3, respectively. Based on the breast GPA scoring system, MOS were found to be 2.3 months for a score of 0–1 (95% CI 1–4.1), 5.7 months for a score of 1.5-2.5 (95% CI 4–8), 10.3 months for a score of 3 (95% CI 8.4-13.7), and 18.4 months for a score > 3 (95% CI 12.4-23.3). The MOS were 21.3 months (95% CI 9.7-53.9) for class I, 9.8 months (95% CI 8.4-12.1) for class II, and 2.3 months (95% CI 1.8-4.3) for class III according to the breast RPA scoring system. Lastly, the MOS for Le Scodan’ scores I, II and III were 15.2 (95% CI 11.5-19.4), 9.7 (95% CI 7.5-12.4), and 4.2 (95% CI 3.3-6.1) months, respectively.

**Table 3:** Distribution of the study population and median overall survival according to the class of prognostic scores; Harrell’s concordance indexes (HCS)

	**Number of pts (%)**	**MOS (95% CI)**	**p**	**Hazard Ratio (95% CI)**	**HCS (95% CI)**
**RPA**					
1	26 (10.4)	25.6 M (18.4–32.9)		1	0.6783
2	166 (66.4)	10.4 M (8.9–12.6)	<0.001	2.16 (1.34–3.50)	(0.65–0.71)
3	58 (23.2)	2 M (1.4–3.1)		11.38 (6.57–19.70)	
**GPA**					
≥3	32 (12.8)	24.7 M (12.7–27.1)		1	0.658
1.5-2.5	116 (46.4)	12.3 M (10.1–15.1)	<0.001	1.70 (1.09–2.65)	(0.62–0.69)
0-1	102 (40.8)	4.2 M (3.1–5.4)		4.15 (2.62–6.55)	
**BS-BM**					
3	31 (12.4)	21.6 M (12.7–25.8)	<0.001	1	
2	68 (27.2)	12.7 M (9.7–18.4)		1.30 (0.81–2.08)	0.6803
1	96 (38.4)	8.7 M (6.1–12.3)		2.20 (1.41–3.46)	(0.64–0.72)
0	55 (22.0)	2.2 M (1.4–3.6)		6.99 (4.25–11.47)	
**P1PS**					
0–1	95 (38.0)	16.4 M (11.9–23.3)		1	0.6251
2–3	64 (25.6)	5.9 M (3.4–8.4)	<0.001	2.89 (2.01–4.14)	(0.58–0.66)
Not available	91 (36.4)				
**Breast GPA**					
3.5-4	53 (21.2)	18.4 M (12.4–23.3)		1	0.6587
3	90 (36.0)	10.3 M (8.4–13.7)	<0.001	1.37 (0.95–2.00)	(0.62–0.69)
1.5-2.5	76 (30.4)	5.7 M (4–8)		2.09 (1.43–3.06)	
0-1	31 (12.4)	2.3 M (1–4.1)		5.75 (3.56–9.27)	
**Breast RPA**					
1	20 (8.0)	21.3 M (9.7–53.9)		1	0.6037
2	192 (76.8)	9.8 M (8.4–12.1)	<0.001	2.05 (1.20–3.50)	(0.57–0.63)
3	38 (15.2)	2.3 M (1.8–4.3)		6.84 (3.69–12.72)	
**Le Scodan Score**					
1	89 (35.6)	15.2 M (11.5–19.4)		1	0.6239
2	49 (19.6)	9.7 M (7.5–12.4)	<0.001	1.32 (0.91–1.92)	(0.58–0.66)
3	112 (44.8)	4.2 M (3.3–6.1)		1.92 (1.43–2.59)	

*CI*, Confidence Interval. *MOS*, Median Overall Survival (from brain metastases diagnosis). *M*, months. *Pts*, patients.

Pairwise comparisons of each PI revealed statistically significant differences in survival between prognostic classes except for the breast GPA classes I vs. II (p = 0.0609), the BS-BM scores 1 vs. 2 (p = 0.27), and Le Scodan’s scores I vs. II (p = 0.098).

### Prognostic indexes comparison

There were no statistically significant differences between all PIs with regard to survival predicting ability (Table 3). Only minor differences were seen using Harrell’s concordance index, with values of Harrell’s C slightly higher for the BS-BM (0.6803) and RPA (0.6783) scoring systems than for the breast RPA (0.6037), Le Scodan’s score (0.6239), and P1PS (0.6251). In multivariate analysis (excluding P1PS whose data were only available for 159 patients), the RPA, Le Scodan’s score, and GPA were found to be the best independent predictors of overall survival. In a second multivariate analysis restricted to the 159 patients with known serum LDH level and proteinemia, the RPA 2 and 3, Le Scodan’s score 3 and P1PS 2/3 were associated with worse survival (Table 4).

**Table 4:** Multivariate Cox regression analyses (stepwise procedure) on (a) general population and (b) population with available biological parameters

**a)**	**HR**	**95% CI**	**P**
**RPA 2**	2.76	1.64–4.66	<0.001
**RPA 3**	8.42	4.36–16.26	<0.001
**Le Scodan 2**	1.49	1.01–2.19	0.041
**Le Scodan 3**	1.87	1.30–2.69	0.001
**GPA 3**	1.75	1.22–2.51	0.002
**b)**	**HR**	**95% CI**	**P**
**RPA 2**	2.42	1.36–4.29	0.003
**RPA 3**	13.26	6.94–25.30	<0.001
**Le Scodan 3**	1.57	1.03–2.39	0.035
**P1PS 2/3**	2.23	1.54–3.24	<0.001

*RPA*, recursive partitioning analysis, *GPA*, graded partitioning analysis, *P1PS*, phase 1 prognostic score, *HR*, hazard ratio, *CI*, confidence interval.

When evaluating the ability of the different scores to correctly stratify patients with short or long life expectancy, the patients with a MOS longer than 12 months accounted for 85%, 75%, 71%, 70%, 68%, 58% and 58% of the “good prognosis” population defined as RPA 1, GPA ≥ 3, BS-BM 3, Breast RPA 1, Breast GPA 3.5-4, Le Scodan 1, BS 0–1, respectively. Patients with a MOS shorter than 3 months accounted for 62%, 39%, 60%, 58%, 61%, 37.5%, and 34% of the “poor prognosis”: population defined as RPA 3, GPA 0–1, BS-BM 0, Breast RPA 3, Breast GPA 0–1, Le Scodan 3, BS 2–3, respectively. The misclassification rates in patients living more than 12 months but classified as “poor prognosis” population were 3%, 17%, 5.5%, 8%, 6.5%, 26% and 25%, respectively. Conversely, the misclassification rates in patients living less than 3 months but classified as “good prognosis” population were 0%, 0%, 3%, 5%, 2%, 4.5% and 7%, respectively.

## Discussion

This comprehensive and simultaneous analysis of 7 prognostic scores was performed on a large, well-characterized and homogeneous population of 250 breast cancer patients with BM. This study examined three common scores, namely the RPA, the GPA, and the BS-BM, as well as four new scores incorporating biological or breast-specific parameters: the breast RPA, the breast GPA, Le Scodan’s score, and the P1PS. With respect to other scoring systems, the Rotterdam score was not investigated since it uses, as a prognostic variable, the clinical response to steroid therapy prior to panencephalic radiotherapy, which is a subjective information not necessarily collected in clinical observations [5]. In the same way, neither the volume of the largest BM, nor the time between BM diagnosis and the beginning of radiotherapy were available to calculate the SIR [6] and Rades [7] scores, respectively.

Until recently, there have been few studies focusing on BM prognostic scores in breast cancer. Yet, it has been demonstrated that the reliability and clinical relevance of these scores vary greatly depending on the type of primary tumor. Sperduto *et al.* found that, in a population of 4,259 patients with 642 breast cancers, the GPA was unfit not only for breast tumor, but also for gastrointestinal, melanoma, and renal cell cancer [8]. Similarly, the widely used RPA index has some limitations in breast disease as it does not consider specific tumor markers, such as the status of HR and HER2. Moreover, the description of extra-cerebral disease is probably not the best suited variable for this pathology, since the prognosis of women with bone metastases or locoregional recurrences differs from that of patients with liver or lung metastases. Recently, efforts have been made to improve accuracy of previous classifications by taking into account breast cancer biomarkers. As such, the GPA score has been replaced by a score specific to breast cancer integrating the status of both HER2 and HR [12]. Likewise, Le Scodan’s score, including the breast cancer molecular subtype and treatment parameters, has been proposed from a retrospective analysis of a selected population of patients presenting with advanced disease [12].

Overall, our results indicated that the different scores were able to discriminate the prognosis of patients, which is in keeping with the analysis of Nieder *et al.* who compared a variety of prognostic classifications from all published trials performed on more than 20 patients [19]. However, the new classifications failed to improve patient selection, with the Breast GPA and Breast RPA scores showing lower Harrell’s concordance indexes than the original RPA score. The diversity of populations between studies might explain discrepancies in results and makes generalization difficult. Indeed, the patients analyzed in the Breast GPA pivotal study did not reflect daily clinical practice since 62% of patients presented 1 to 3 BM, 35% had BM without extra-cranial metastases, 37% were aged less than 50 years, 57% had tumors overexpressing HER2 receptor, and 68% of patients received targeted local treatments, which probably explains an impressively good survival (13.8 months). Regarding the results from the Breast RPA pivotal study, in comparison of our study population, the irradiation of 98% of the population represents a selection bias related to the treatment received after BM diagnosis compared to a general clinical practice situation [14]. Contrary to previous indexes, Le Scodan’s score had an independent prognostic value in multiparametric analysis, emphasizing the importance of biological subtypes and blood parameters [13]. However, the drawback is that the definition of biological subtype varies depending on the author. Le Scodan *et al.* distinguished between HER2 positive population treated with trastuzumab and triple negative breast cancer [13], while Sperduto *et al.*[12] and Niwinska *et al.*[14] distinguished between luminal A, B, HER2, and basal tumors. In these last two studies, 77% and 50% of the HER2+ population were treated using anti-HER2 agents, respectively. It would have been interesting to integrate, as did Le Scodan, the anti-HER2 treatment in the biological subtype since there is increasing evidence that anti-HER2 treatments prolong survival of breast cancer patients with BM [9-11,20]. Biological parameters, such as lymphopenia for Le Scodan’s score and LDH and proteinemia for the P1PS [4], have been shown to have an independent prognostic value on multiparametric analysis and thus warrant further evaluation. Evaluating subclinical disease activity and the impact on nutritional status may confer additional prognostic information.

One of the strengths of our study is to reflect routine clinical practice population, without selection based on performance status, number of metastases or treatment. This is essential to provide physicians with a clinical tool applicable to the whole patient population at the time of BM diagnosis. According to our analysis, the RPA score can still be considered as the reference score for several reasons. Firstly, although Harrell’s concordance Indexes were quite similar for all PIs, the hazard ratio of the RPA was higher than those of other PIs in multivariate analysis. Our results were consistent with those reported by (i) Le Scodan *et al.*[21] and Mahmoud-Ahmed *et al.*[22] who confirmed the prognostic value of the RPA score in the setting of BM from breast cancer (ii) Viani *et al.* who found a superiority of the RPA score over the BS-BM one [23]. Secondly, one must keep in mind the primary goal of these classifications which is to adapt treatment options to the individual patient prognosis. We need to mitigate the treatment burden for patients with short life expectancy, and conversely to intensify therapeutic interventions for patients for whom an improvement in overall survival is expected. Hence, it is important to know how often the prognostic scores wrongly categorize patients in inappropriate prognosis groups. Nieder *et al.* studied their ability to correctly classify patients with good prognosis (MOS longer than 6 months from the diagnosis of BM) and patients with poor prognosis (MOS shorter than 2 months from the diagnosis of BM) [24]. In our study, the MOS was 8.9 months and 40% of the population was alive at 1 year, so we decided to adapt the cut offs used by Nieder to our study population, and we considered boundaries to be a MOS of less than 3 months and a MOS of more than 12 months. In these circumstances, the RPA proved to be more efficient than the other scores to predict median survival since 85% of patients classified as RPA 1 survived more than 12 months, and 62% of patients classified as RPA 3 survived less than 3 months. Furthermore, the RPA misclassified a smaller proportion of patients than the other scoring systems as no patients classified RPA 1 survived less than 3 months and only 3% of patients classified as RPA 3 survived more than 12 months.

A particular weakness of some of the classification systems is the lack of homogeneous distribution of patients between the different prognostic categories. Indeed, a score that would identify a subgroup with excellent prognosis in a very small number of patients, a situation rarely seen in clinical practice, would have limited impact to aid therapeutic decision making in routine practice. This is one of the pitfalls of the GPA scoring since the class 3.5-4 of better prognosis accounts only for 2.8% of our daily clinical practice population. Finally, an ideal prognostic score should be simple and easily usable in clinical practice. Our analysis at this stage differs from that of Sperduto *et al.*[2] in so far that we believe that the RPA score is more readily reproducible in practice thanks to a limited number of variables to be collected and fewer prognostic classes.

Nevertheless, due to its retrospective nature, our study suffers some limitations. First, in retrospective analysis, it could be difficult to assess controlled versus uncontrolled distant metastases. As this information is required in Breast RPA prognostic index, the retrospective analysis of this factor could have misclassified some patients. Similarly, a retrospective evaluation of KPS appears less reliable than the evaluation of Performance Status using ECOG classification, and could have led to some degrees of misclassification.

## Conclusion

The new PIs did not perform better than the original scores. Although tumor subtypes, HER2 expression, and blood parameters (LDH, proteinemia, lymphopenia) may have an interesting additional prognostic value, the RPA appears to be the most appropriate and simplest available tool to help clinicians select breast cancer patients with BM.

## Abbreviations

BM: Brain metastases; BS-BM: Basic score for brain metastases; GPA: Graded prognostic assessment; HER2: Human epidermal growth factor receptor-2; HR: Hormonal receptor; IHC: Immunohistochemistry; KPS: Karnofsky performance status; LDH: Lactate dehydrogenase; MOS: Median overall survival; OS: Overall survival; P1PS: Phase 1 prognostic score; PI: Prognostic indexes;RPA: Recursive partitioning analysis; SIR: Score index for radiosurgery; ULN: Upper limit of normal.

## Competing interests

The authors declare that they have no conflict of interest.

## Authors’ contributions

Conception and design: ALB, WJ, D. Provision of study material or patients: ALB, WJ, DA, J-MF, GR. Collection and assembly of data: ALB, WJ, ST. Data analysis and interpretation: ALB, WJ, DA, ST. Manuscript writing: ALB, WJ, DA, ST. Final approval of the manuscript: ALB, WJ, DA, J-MF, GR, ST.

## Pre-publication history

The pre-publication history for this paper can be accessed here:

http://www.biomedcentral.com/1471-2407/13/70/prepub
